# Microcystin-LR Induces Apoptosis via NF-κB/iNOS Pathway in INS-1 Cells

**DOI:** 10.3390/ijms12074722

**Published:** 2011-07-22

**Authors:** Yong Ji, Gao Lu, Guoqiang Chen, Bin Huang, Xian Zhang, Kai Shen, Song Wu

**Affiliations:** 1 Department of Cardiothoracic Surgery, The Affiliated Jiangyin People’s Hospital of Southeast University Medical College, No.163 Shoushan Road, Jiangyin 214400, Jiangsu, China; E-Mails: ga1984ag@126.com (Y.J.); GuoQiangChen_nj@163.com (G.C.); BinHuang_nj@yeah.net (B.H.); XianZhang_nj@126.com (X.Z.); KaiShen_nj@126.com (K.S.); 2 Key Laboratory of Human Functional Genomics of Jiangsu Province, Nanjing Medical University, Nanjing 210029, Jiangsu, China; E-Mail: gaolu_nj@126.com

**Keywords:** INS-1, NF-κB, iNOS, Microcystin-LR, apoptosis

## Abstract

Cyanobacterial toxins, especially the microcystins, are found in eutrophied waters throughout the world, and their potential to impact on human and animal health is a cause for concern. Microcystin-LR (MC-LR) is one of the common toxic microcystin congeners and occurs frequently in diverse water systems. Recent work suggested that apoptosis plays a major role in the toxic effects induced by MC-LR in hepatocytes. However, the roles of MC-LR in pancreatic beta cells have not been fully established. The aim of the present study was to assess possible *in vitro* effects of MC-LR on cell apoptosis in the rat insulinoma cell line, INS-1. Our results demonstrated that MC-LR promoted selectively activation of NF-κB (increasing nuclear p50/p65 translocation) and increased the mRNA and protein levels of induced nitric oxide synthase (iNOS). The chronic treatment with MC-LR stimulated nitric oxide (NO) production derived from iNOS and induced apoptosis in a dose dependent manner in INS-1 cells. Meanwhile, this effect was inhibited by the NF-κB inhibitor PDTC, which reversed the apoptosis induced by MC-LR. Our observations indicate that MC-LR induced cell apoptosis via an iNOS-dependent pathway. A well-known nuclear transcription factor, NF-κB, is activated and mediates intracellular nitric oxide synthesis. We suggest that the apoptosis induced by chronic MC-LR *in vivo* presents a possible cause of β-cell dysfunction, as a key environmental factor in the development of diabetes mellitus.

## 1. Introduction

Microcystins (MCs) are a group of closely related cyclic heptapeptides produced by a variety of common cyanobacteria. These are potent and highly specific hepatotoxins, the toxicity of which is based upon their inhibition of protein phosphatase 1 and 2A, resulting in hyperphosphorylation of cytoskeletal proteins [1]. Various other MC effects have also been reported, including neurotoxicity, genotoxicity, and embryotoxicity, with some studies elucidating the potency of MCs as immune intruders [2]. The adverse effects of MC-LR, as a key member of the microcystins, are closely related to oxidative stress processes, free radicals and DNA damage, involved in major gene transcript changes [3,4]. MC-LR induces programmed cell death or apoptosis both *in vivo* and *in vitro* [3,5]. However, the exact mechanisms underlying the suggested apoptosis-inducing potential are still unknown. The expression of p53, Bcl-2 and Bax are involved in the regulation of MC-LR induced apoptosis [6]. Recent studies have confirmed that the mechanism of MC-LR-induced hepatocyte apoptosis is thought to include the involvement in oxidative stress [3,7]. In addition, NF-κB mediates cellular apoptosis by MC-LR in HepG2 cells [8]. When human melanoma cells are exposed to exceptionally high concentrations of the toxins, binding to the ATP synthetase subunit could be a trigger of mitochondrial apoptotic signaling through perturbation of mitochondrial functions, with possible leakage of cytochrome [9]. Apoptosis plays a major role in the toxicity induced by microcystin-LR (MC-LR) *in vivo*.

The transcription factor, nuclear factor kappa B (NF-κB), is identified as a protein that binds to a specific DNA sequence, which is central in the regulation of inflammatory responses and control of the innate immune system [10]. The general term NF-κB traditionally refers to the p50/p65 (p50/RelA) heterodimer, which is an apoptotic gene regulator. NF-κB**-**p65, a subunit of the NF-kappa-B transcription complex, provides the gene regulatory function and plays a crucial role in inflammatory and immune responses [11]. The inhibitory effect of I-kappa-B upon NF-kappa-B in the cytoplasm is exerted primarily through the interaction with p65. p65 shows a weak DNA-binding site which could contribute directly to DNA binding in the NF-kappa-B complex. The role of NF-kB in apoptosis is, up to now, still controversial. In fact, it has been reported that it is either independent [12], or required in both insulin-producing cells and in tumor cell lines [13,14]. NF-κB, as important transcription factor, mediates various biological functions *in vivo*, but not as a final effector molecule. Previous studies revealed that NF-κB acts in synergy with other transcription factors such as Ap-1 or Sp1 in order to mediate an effective transcriptional activation. This suggests that a distinct combination of binding sites for different transcription factors within individual gene promoters contributes to the selective regulation of gene expression [15].

Nitric oxide (NO) is now recognized as one of the most important molecules influencing the development, progression and treatment of diseases. A key component of its action is as a negative and positive regulator of apoptosis [16]. Constitutive levels of NO can be cytoprotective, in some instances by acting as a free radical-scavenging antioxidant [17]. Many tumors produce low constitutive levels of NO, which has been reported to promote angiogenesis and tumor growth [18]. During infection, NO plays a crucial role in host defense mechanisms [19]. Rapid induction of iNOS expression can trigger NO-dependent apoptosis *in vitro*, which appears to result from DNA damage and may be mediated by a p53-dependent apoptotic pathway [20]. iNOS expression is typically absent in unstimulated cells, but is markedly induced by pro-inflammatory cytokines including tumor necrosis factor-a, interleukin-1β, and interleukin-6 [21,22]. Previous studies have shown that NO is an important modulator of apoptosis in islet cells. Observations in islet cells have shown that IL-1β-mediated toxicity is partly induced by production of reactive oxygen species, in particular nitric oxide (NO), through the activation of the NF-κB pathway [23]. iNOS gene silencing also protects these cells from inflammatory cytokine-induced apoptosis and increases their capacity to secret insulin [24]. Therefore, intracellular NO plays an important role in apoptosis in islet cells.

Based on the results described above, the aim of the present study was to investigate the toxicity of MC-LR on pancreatic beta cell using the beta cell line INS-1.

## 2. Material and Methods

### 2.1. Reagents

Microcystin-LR (HLPC content 98%) was purchased from Alexis Biochemicals, Switzerland. Ammonium pyrrolidine dithiocarbamate (PDTC, p8765) was purchased from Sigma-Aldrich, USA.

RPMI-1460 medium and Fetal bovine serum (FBS) were purchased from Gibco, USA. The Lipofectamine 2000 reagent was obtained from Invitrogen Life Technologies, USA. The luciferase reporter assay system and avian myeloblastosis virus reverse transcription system were obtained from Promega, USA. The rabbit polyclonal antibody against iNOS was purchased from Santa Cruz Biotechnology, USA. The rabbit polyclonal antibodies against p65 and p53 were purchased from Cell signal technology, USA. Double Stain Apoptosis Detection Kit (Hoechst 33342/PI) was purchased from GenScript, USA. Nitric Oxide Assay Kit (Griess Reagent) was purchased from Beyotime, CHINA.The Annexin-V-FITC Apoptosis Detection Kit was purchased from BD Biosciences, USA.

### 2.2. Cell Culture

INS-1, a rat insulinoma cell line, was obtained from American type culture collection (ATCC), USA. The cells were grown in RPMI-1640 medium supplemented with 10% fetal bovine serum (FBS), 10 mmol/L HEPES, 2 mmol/L L-glutamine, 50 μmol/L β-mercaptoethanol, 1 mmol/L sodium pyruvate, 100 U/mL penicillin, and 100 μg/mL streptomycin at 37 °C in a humidified atmosphere containing 95% air and 5% CO_2_. The cells were exposed to various concentrations (50~1000 nM) of MC-LR or to PDTC (1 μM or 5 μM) for the indicated times. Before the co-treatment with MC-LR and PDTC, cells were pretreated with PDTC for 2 h.

### 2.3. MTT Assay

Cell viability was determined using a 3-(4,5-dimethylthiazol-2-yl)-2,5-diphenyltetrazolium bromide (MTT) assay. Briefly, the cells were seeded in 96-well dishes at 1 × 10^4^ to 2 × 10^4^ cells per well, and pretreated with or without MC-LR for 72 h. Each well was then supplemented with 10 μL MTT (Sigma) and incubated for 4 h at 37 °C. The medium was then removed, and 150 μL dimethyl sulfoxide (Sigma) was added to solubilize the MTT formazan. The optical density was read at 490 nm.

### 2.4. Hoechst/PI Staining

Apoptotic cell death was evaluated by staining the non-viable cells red with propidium iodide (PI) and Hoechst 33342 (Sigma), which stained the nuclei of both live and dead cells blue. Staining with Hoechst allows for the discrimination of apoptotic cells on the basis of nuclear morphology and evaluation of membrane integrity. The Hoechst dye was added to the culture medium at a final concentration of 5 μg/mL, and the cultured cells were incubated at 37 °C for 30 min. The PI (5 μg/mL) solution was then also added immediately before observation in the fluorescence microscope.

### 2.5. Flow Cytometry

To estimate the number of apoptotic cells, cells were fluorescently labeled by addition of 20 μL of binding buffer, 5 μL of Annexin V-FITC and 5 μL of propidium iodide. After incubation at room temperature in the dark for 15 min, cells were applied to flow cytometry analysis. A minimum of 10,000 cells in the gated region was analyzed using a BD FACS Calibur Flow Cytometer. Results were interpreted by the percentage of total cells appearing in each quadrant.

### 2.6. Immunofluorescence Microscopy

A standard immunostaining procedure was carried out to observe NF-κB nuclear translocation activity. Cells grown on thick slides were washed with PBS, fixed by immersion at room temperature with 4% polyformaldehyde for 20 min, and permeabilized with 0.1% Triton-X-100 in PBS at 4 °C for 10 min. Slides were then washed with PBS and blocked with blocking buffer consisting of 4% bovine serum albumin (BSA) in PBS for 30 min at room temperature and then incubated with primary anti-NF-κB (p65) monoclonal antibody diluted 1:25 in blocking buffer overnight at 4 °C, followed by a secondary anti-rabbit FITC-labeled antibody incubation diluted 1:100 in blocking buffer at room temperature for 1h. Subsequently, cells were stained with 5 μg/mL DAPI for 2 min and washed with PBS. Coverslips and stained cells were captured and analyzed by a confocal laser scanning microscopy system using a 400× magnification (LSM710, Carl Zeiss, Germany). Control samples with HRP-conjugated secondary antibody and no DAPI showed a faint background staining (data not shown).

### 2.7. Transient Transfection and Luciferase Reporter Assay

The luciferase reporter construct 3×NF-κB-LUC and 3 × AP-1-LUC(NF-κB/AP-1 responsive elements) was transiently transfected into INS-1 cells grown in 24 well plates using the Lipofectamine 2000 reagent according to the manufacturer’s instructions. The luciferase reporter construct driven by three copies of the NF-κB or AP-1 response elements were from B. M. Forman (Department of Gene Regulation and Drug Discovery, Beckman Research Institute of City of Hope National Medical Center, Duarte, CA, USA). A plasmid expressing the gene encoding β-galactosidase driven by the cytomegalovirus (CMV) promoter (Clontech Laboratories, Palo Alto, CA, USA) was simultaneously cotransfected as an internal control. The medium was replaced 4 h after transfection. Twenty-four hours after transfection, the cells were treated with the indicated concentrations of MC-LR or PDTC for an additional 24 h and harvested for luciferase reporter assays as described previously [25].

### 2.8. Real-Time RT-PCR Assay

Cells were cultured and treated as described above. The total RNA was extracted using Trizol reagent. First-strand cDNA synthesis was performed using 1 μg of total RNA and an avian myeloblastosis virus reverse transcription system. The primers were designed using primer express software (Applied Biosystems, Foster City, CA, USA). Real-time quantitative PCR was performed using the SYBR Green PCR Master Mix and ABI Prism 7300 Sequence Detection System (Applied Biosystems). All data were analyzed using the expression of the gene encoding β-actin as a reference. The sequences of the primers used are available upon request. The iNOS gene primers were as follows: forward primer, 5′-CTCACTGTGGCTGTGGTCACCTA-3′ and reverse primer, 5′-GGGTCTTCGGGCTTCAGGTTA-3′. The relative expression levels were calculated according to the formula 2^−ΔCt^, where ΔCt is the difference in threshold cycle (Ct) values between the target gene and the endogenous control.

### 2.9. Western Blot

Cells were cultured and treated as described above, and then lysed with ice-cold lysis buffer containing 50 mmol/L Tris-HCl, pH 7.4; 1% NP-40; 150 mmol/L NaCl; 1 mmol/L EDTA; 1 mmol/L phenylmethylsulphonyl fluoride; and complete proteinase inhibitor mixture (one tablet per 10 mL; Roche Molecular Biochemicals, Indianapolis, IN, USA). After protein content determination using a DC Protein Assay kit (Bio-Rad Laboratories, Hercules, CA, USA), Western blotting was performed as described previously [26].

### 2.10. NO Assay

INS-1 cells were seeded into 48-well plates for 24 h, and media was subsequently replaced by 200 μL of serum-free medium per well ± MC-LR. After 48 h of incubation, the medium was sampled for NO determination using the Griess method.

### 2.11. Statistical Analysis

Differences between groups were analyzed using two-sided *t* test and ANOVA with p < 0.05 considered statistically significant.

## 3. Results

### 3.1. MC-LR Selectively Promotes Activation of NF-κB in INS-1 cells

**Figure 1:**
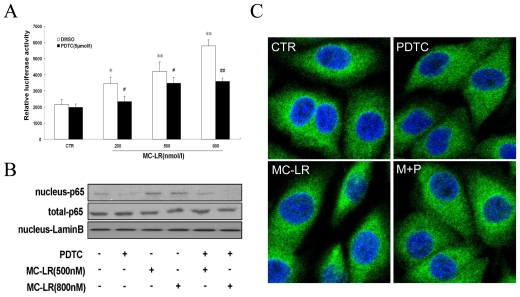
Microcystin-LR (MC-LR) induces NF-κB activation in INS-1 cells. Cells were treated with the indicated concentrations of MC-LR and PDTC (5 μmol/L) or not for 24 h. (**A**) MC-LR dose-dependently increased NF-κB activation in luciferase reporter assay. Values are the means ± SD (n = 3) of three individual experiments. *p < 0.05, **p < 0.01 *vs*. control (DMSO); ^#^p < 0.05, ^##^p < 0.01 *vs*. group at the same dose of MC-LR without PDTC; (**B**) Protein level of the p65 subunit of NF-κB, as determined by Western blot; (**C**) Representative immunofluorescence localization for p65 subunit. MC-LR increased NF-κB translocation from the cytoplasm to the nucleus. All MC-LR-induced increases were reversed by PDTC.

Nuclear factor kappa B (NF-κB) and activator protein 1 (AP-1) transcription factors regulate many important biological and pathological processes. Activation of NF-κB is mainly regulated by pro-inflammatory cytokines and bacterial toxins (e.g., LPS, exotoxin B) [27]. AP-1 is a transcription factor which is a heterodimeric protein composed of proteins belonging to the c-Fos, c-Jun, ATF and JDP families. Fos, a key component of AP-1, is primarily transcriptionally regulated by various stimuli, including cellular stress, ionizing and ultraviolet irradiation, DNA damage, and oxidative stress [28,29]. To analyze the relationship between nuclear transcription factor activity and MC-LR, activation of NF-κB and AP-1 was determined by luciferase reporter assay. In our studies, MC-LR had no significant effect on activation of AP-1 (data not shown). As depicted in Figure 1A, MC-LR dose-dependently increased NF-κB response element reporter gene activity (NF-κB DNA-binding activity) in cells, with maximum induction occurring at a concentration of 800 nmol/L (increases of 268% *vs*. control, p < 0.01). Furthermore, MC-LR increased NF-κB translocation from the cytoplasm to the nucleus, increasing protein level of nuclear NF-κB (p65) as evaluated by Western blot and immunostaining (Figure 1B and 1C). These data suggest that NF-κB, but not AP-1, is a mediator for MC-LR -initiated toxicity in INS-1 cells.

### 3.2. MC-LR Up-Regulates iNOS Expression and Stimulates NO Formation in INS-1 Cells

**Figure 2:**
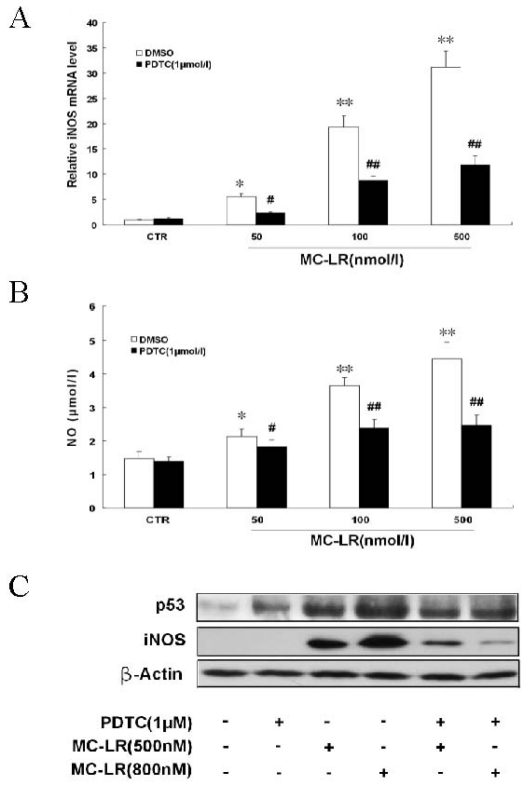
MC-LR accelerates intracellular nitric oxide (NO) production by up-regulating iNOS expression. Cells were treated with the indicated concentrations of MC-LR and PDTC (1 μmol/L) for 48 h. (**A**,**B**). MC-LR significantly induced iNOS mRNA level and increased NO synthesis, and PDTC partly inhibited this effect. Values are the means ± SD (n = 3) of three individual experiments. *p < 0.05, **p < 0.01 *vs*. control (DMSO); ^#^p < 0.05, ^##^p < 0.01 *vs*. group at the same dose of MC-LR without PDTC; (**C**) MC-LR up-regulated iNOS and p53 protein expression in a dose-dependent manner.

iNOS expression plays a critical role in proinflammatory cytokine-induced NO production [30]. In order to determine whether MC-LR accumulated NO production by up-regulation of iNOS expression, we investigated the mRNA and protein levels of iNOS by real-time RT-PCR and Western blot. As shown in Figure 2, iNOS transcription and translation were markedly increased when cells were treated with MC-LR for 48 h. Moreover, PDTC attenuated MC-LR-induced changes of iNOS mRNA and protein. Extracellular NO in the media from INS-1 cells was not markedly detected until treatment with a low concentration of MC-LR for 48 h (Figure 2B). NO is known to up-regulate and activate p53 (Cook *et al*., 2004; Qiu *et al*., 2004) [31,32]. NO accumulation also accounts for the p53-dependent apoptotic response to DNA damage. To evaluate whether NO formation mediated cell apoptosis, the expression of p53 was observed by Western blot, which showed that the induction of iNOS coincided with p53 up-regulation when cells were treated with MC-LR for 48 h (Figure 2C). Therefore, NO derived from iNOS plays an important role in MC-LR-induced β-cell toxicity.

### 3.3. Chronic Treatment with MC-LR Induces Apoptosis in INS-1 Cells

**Figure 3:**
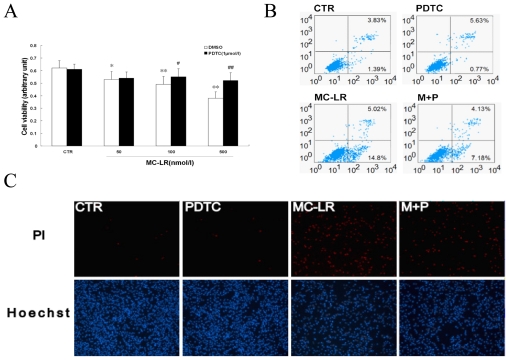
MC-LR induces apoptosis in INS-1 cells. (**A**) Cells were treated with the indicated concentrations of MC-LR and PDTC (1 μmol/L) for 72 h, analyzed by MTT assay. Values are the means ± SD (n = 3) of three individual experiments. *p < 0.05, **p < 0.01 *vs*. control (DMSO); ^#^p < 0.05, ^##^p < 0.01 *vs*. group at the same dose of MC-LR without PDTC. Cells were treated with MC-LR (500 nmol/L) and PDTC (1 μmol/L) for 72 h; (**B**) Cells were stained with Annexin V-FITC and PI, analyzed by flow cytometry. Data are expressed as % of Annexin V-FITC-positive and PI-negative cells (early stage of apoptosis) and as % of Anexin V-FITC- and PI-positive cells (late stage of apoptosis and necrosis); (**C**) Representative photographs of double staining of PI and Hoechst 33342. The apoptotic cells were observed as PI intense signal after double staining.

Maintenance of beta cell mass is critical for secretion of adequate amounts of insulin. Dysfunction induced by the decreased population of cells is regarded as an important factor in the pathogenesis of various metabolic diseases. To investigate the effect of MC-LR on pancreatic beta cells, cell viability was determined using MTT assays. MC-LR inhibited the viability of INS-1 cells in a dose-dependent manner (Figure 3A). To further examine the effects of MC-LR on pancreatic beta cell growth, two different methods were used. Determination of DNA content by Hoechst/PI staining indicated that MC-LR increased the amount of dead cells in INS-1 cells (Figure 3C). Quantitative evaluation of apoptosis through annexin V-FITC/PI staining was analyzed by Flow Cytometry. As shown in Figure 3B, the rate of apoptotic cells raised to 19.82% with the treatment of MC-LR (500 nmol/L) for 72 h. Furthermore, pretreatment with PDTC prevented MC-LR-induced apoptosis, which was demonstrated in the results described above. It seemed that NF-κB played a major role in apoptosis mediated by MC-LR *in vitro*. Taken together, these results suggest that MC-LR activated the NF-κB signaling pathway to induce cells apoptosis.

## 4. Discussion

Microcystin-LR (MC-LR), a potent environmental hepatotoxin produced by blue-green algae in eutrophic surface waters, has received increasing worldwide attention in recent decades. In recent years, the effect of Microcystins was mainly focused on liver injury *in vivo*. It was confirmed that MC-LR could induce the production of large amounts of ROS in primary hepatocytes [3,33], and stimulated the sustained activation of JNK (C-Jun N-terminal kinase) and its downstream targets [34]. Some results showed that MC-LR could induce apoptosis in a variety of cell types, characterized by cell membrane blebbing, cytoplasmic shrinkage, nuclear chromatin condensation, DNA fragmentation and formation of apoptotic bodies. In addition, MC-LR could affect Ca^2+^-channels and insulin release by inhibiting an extracellular phosphatase-like activity [35]. However, the role of Microcystins in the regulation of pancreatic beta cell apoptosis is not clear. Our results provided new insight into MC-LR toxicity. MC-LR promoted NF-κB activation and increased NO accumulation mediated by NF-κB. We speculated that the NO-dependent apoptosis might be the main toxic pathway for MC-LR in INS-1 cells instead of inhibition to protein phosphatase activity. Apoptosis had been evidenced indirectly by three independent methods in our studies. Furthermore, it has been reported that the loss of β-cells observed late in the course of diabetes mellitus might be the result of NO-induced apoptosis [36]. Therefore, Microcystin-LR, as an environmental factor, possibly plays an important role in the development of diabetes.

It is well-known that pancreatic β-cell dysfunction is caused by chronic multi-factors such as gluco-lipotoxicity and inflammatory mediators [37,38]. It has been reported that the β-cell decompensation in the form of diabetes may involve excess nitric oxide generation by free fatty acids [39]. Glucose induced production of IL-1beta in β-cells contributes to glucotoxicity in human pancreatic islets. Moreover, *in vitro* exposure of islets from non-diabetic organ donors to high glucose levels resulted in increased production and release of IL-1β, followed by NF-κB activation, iNOS up-regulation, DNA fragmentation, and impaired β-cell function [40]. Thus, the islet expression of iNOS seems to be, at least in part, a common signal pathway in both lipotoxicity and glucotoxicity. In addition, IL-1β may be produced by beta cells themselves or by homing macrophages within the pancreas. Islet inflammation and increased beta cell death have in particular been associated with the cytokine IL-1β [40]. Therefore, NO is a key marker of the signal transduction pathway for apoptosis in islet cells. The mechanism of apoptosis induced by MC-LR showed such similarity in INS-1 cells. We found that MC-LR up-regulated the mRNA level of iNOS by more than 30-fold in β-cells (Figure 2A). MC-LR-induced NO accumulation was shown in a time and dose dependent manner, with maximum production after 48 h (Figure 2B). Furthermore, MC-LR induced iNOS transcription, radical formation and β-cell death were all reversed by NF-κB inhibitor, indicating a link between NO accumulation and β-cell dysfunction.

Nuclear factor kappa B (NF-κB) is a transcription factor thought to play an important role in the onset of cell apoptosis. NF-κB activation coincided with the expression of the pro-inflammatory marker iNOS. To determine the effects of NF-κB inhibition during apoptosis, the NF-κB inhibitor pyrrolidine dithiocarbamate (PDTC) was given. The apoptosis from MC-LR to islets mediated by iNOS could be suppressed by PDTC, which led to cell survival (Figure 3). For this reason, NF-κB might become a candidate target for new anti-inflammatory and anti-apoptosis treatment. However, the abrogation of NF-κB activation did not completely prevent INS-1 cells from MC-LR-induced apoptosis, suggesting that this event may not be totally dependent on NF-κB activation.

Microcystin is a cyclic peptide comprising seven amino acids, as a potent hepatotoxin produced by cyanobacteria. Only three human proteins (organic anion-transporting polypeptides OATP1B1, OATP1B3, and OATP1A2) are thought to be able to mediate the hepatic uptake of microcystins, and the predominant hepatic expression of these transporters accounts for the liver-specific toxicity of microcystins [41]. A significant obstacle in the study of microcystins is the requirement of specific transport proteins for cellular uptake. It has been reported that these transporters are expressed in a few cell lines created from liver, colon, and pancreatic tumors [42,43]. It was suggested that MC-LR induced apoptosis *in vitro* with obvious individual difference. Our observations indicated that a series of β-cell lines presented different sensitivities to apoptosis induced by MC-LR, which was observed to be very resistant in MIN6 cells compared with INS-1 cells. In addition, MC-LR inhibited the viability of INS-1 cells at the indicated concentrations (Figure 3A) as determined by MTT assay, but MC-LR had no significant effect on apoptosis at the same dose (50 nmol/L) as analyzed by Hoechst/PI staining and flow cytometry (data not shown). When the concentration of MC-LR was increased to 500 nmol/L, apoptosis was markedly increased (Figure 3B and 3C). These results suggested that MC-LR might inhibit pancreatic islet beta cell proliferation partly through cell apoptosis.

The uptake amount of MC-LR in mammals from drinking water was difficult to detect [44]. For this reason, the effect of chronic toxicity was constantly dismissed. When cells were exposed to MC-LR (10 μmol/L, beyond the physiological range) for 16 h, apoptosis was rapidly induced (data not shown). However, MC-LR was able to induce apoptosis when cells were treated for a long period of time at lower concentrations (Figure 3), which corresponded with toxicity *in vivo*. Therefore, a low concentration of MC-LR in water could significantly interrupt cellular processes, and more care should be taken in determining the criteria for microcystins content in drinking water.

In summary, we have demonstrated that MC-LR activated the NF-κB signaling pathway to up-regulate iNOS expression and induce cells apoptosis, which provides additional insight into the mechanism of MC-LR-induced toxicity *in vivo*.

## References

[1] Honkanen RE, Zwiller J, Moore RE, Daily SL, Khatra BS, Dukelow M, Boynton AL (1990). Characterization of microcystin-LR, a potent inhibitor of type 1 and type 2A protein phosphatases. J. Biol. Chem.

[2] Chen T, Shen P, Zhang J, Hua Z (2005). Effects of microcystin-LR on patterns of iNOS and cytokine mRNA expression in macrophages *in vitro*. Environ. Toxicol.

[3] Weng D, Lu Y, Wei Y, Liu Y, Shen P (2007). The role of ROS in microcystin-LR-induced hepatocyte apoptosis and liver injury in mice. Toxicology.

[4] Lankoff A, Krzowski L, Glab J, Banasik A, Lisowska H, Kuszewski T, Gozdz S, Wojcik A (2004). DNA damage and repair in human peripheral blood lymphocytes following treatment with microcystin-LR. Mutat. Res.

[5] McDermott CM, Nho CW, Howard W, Holton B (1998). The cyanobacterial toxin, microcystin-LR, can induce apoptosis in a variety of cell types. Toxicon.

[6] Brzuzan P, Wozny M, Ciesielski S, Luczynski MK, Gora M, Kuzminski H, Dobosz S (2009). Microcystin-LR induced apoptosis and mRNA expression of p53 and cdkn1a in liver of whitefish (Coregonus lavaretus L.). Toxicon.

[7] Botha N, Gehringer MM, Downing TG, van de Venter M, Shephard EG (2004). The role of microcystin-LR in the induction of apoptosis and oxidative stress in CaCo_2_ cells. Toxicon.

[8] Feng G, Abdalla M, Li Y, Bai Y (2011). NF-kappaB mediates the induction of Fas receptor and Fas ligand by microcystin-LR in HepG2 cells. Mol. Cell. Biochem.

[9] Mikhailov A, Harmala-Brasken AS, Hellman J, Meriluoto J, Eriksson JE (2003). Identification of ATP-synthase as a novel intracellular target for microcystin-LR. Chem. Biol. Interact.

[10] Tak PP, Firestein GS (2001). NF-kappaB: A key role in inflammatory diseases. J. Clin. Invest.

[11] Czyz M (2005). Specificity and selectivity of the NFkappaB response. Postepy. Biochem.

[12] Velez-Pardo C, Morales AT, Del-Rio MJ, Olivera-Angel M (2007). Endogenously generated hydrogen peroxide induces apoptosis via mitochondrial damage independent of NF-kappaB and p53 activation in bovine embryos. Theriogenology.

[13] Darville MI, Eizirik DL (2001). Cytokine induction of Fas gene expression in insulin-producing cells requires the transcription factors NF-kappaB and C/EBP. Diabetes.

[14] Wurzer WJ, Ehrhardt C, Pleschka S, Berberich-Siebelt F, Wolff T, Walczak H, Planz O, Ludwig S (2004). NF-kappaB-dependent induction of tumor necrosis factor-related apoptosis-inducing ligand (TRAIL) and Fas/FasL is crucial for efficient influenza virus propagation. J. Biol. Chem.

[15] Dvorak Z, Pavek P (2008). Comment on “The role of redox-sensitive transcription factors NF-kB and AP-1 in the modulation of the Cyp1A1 gene by mercury, lead, and copper”. Free Radic. Biol. Med.

[16] Kong G, Kim E, Kim W, Lee Y, Lee J, Paik S, Rhee J, Choi K, Lee K (2001). Inducible nitric oxide synthase (iNOS) immunoreactivity and its relationship to cell proliferation, apoptosis, angiogenesis, clinicopathologic characteristics, and patient survival in pancreatic cancer. Int. J. Gastrointest Cancer.

[17] Wink DA, Mitchell JB (1998). Chemical biology of nitric oxide: Insights into regulatory, cytotoxic, and cytoprotective mechanisms of nitric oxide. Free Radic. Biol. Med.

[18] Siamwala JH, Majumder S, Tamilarasan KP, Muley A, Reddy SH, Kolluru GK, Sinha S, Chatterjee S (2010). Simulated microgravity promotes nitric oxide-supported angiogenesis via the iNOS-cGMP-PKG pathway in macrovascular endothelial cells. FEBS Lett.

[19] Gookin JL, Chiang S, Allen J, Armstrong MU, Stauffer SH, Finnegan C, Murtaugh MP (2006). NF-kappaB-mediated expression of iNOS promotes epithelial defense against infection by cryptosporidium parvum in neonatal piglets. Am. J. Physiol. Gastrointest Liver Physiol.

[20] Tian B, Liu J, Bitterman PB, Bache RJ (2002). Mechanisms of cytokine induced NO-mediated cardiac fibroblast apoptosis. Am. J. Physiol. Heart Circ. Physiol.

[21] Corbett JA, Kwon G, Misko TP, Rodi CP, McDaniel ML (1994). Tyrosine kinase involvement in IL-1 beta-induced expression of iNOS by beta-cells purified from islets of Langerhans. Am. J. Physiol.

[22] Medeiros R, Prediger RD, Passos GF, Pandolfo P, Duarte FS, Franco JL, Dafre AL, Giunta G, Figueiredo CP, Takahashi RN (2007). Connecting TNF-alpha signaling pathways to iNOS expression in a mouse model of Alzheimer’s disease: Relevance for the behavioral and synaptic deficits induced by amyloid beta protein. J. Neurosci.

[23] Gurgul E, Lortz S, Tiedge M, Jorns A, Lenzen S (2004). Mitochondrial catalase overexpression protects insulin-producing cells against toxicity of reactive oxygen species and proinflammatory cytokines. Diabetes.

[24] Li F, Mahato RI (2008). iNOS gene silencing prevents inflammatory cytokine-induced beta-cell apoptosis. Mol. Pharm.

[25] Han X, Sun Y, Scott S, Bleich D (2001). Tissue inhibitor of metalloproteinase-1 prevents cytokine-mediated dysfunction and cytotoxicity in pancreatic islets and beta-cells. Diabetes.

[26] Meng ZX, Nie J, Ling JJ, Sun JX, Zhu YX, Gao L, Lv JH, Zhu DY, Sun YJ, Han X (2009). Activation of liver X receptors inhibits pancreatic islet beta cell proliferation through cell cycle arrest. Diabetologia.

[27] Song JD, Lee SK, Kim KM, Kim JW, Kim JM, Yoo YH, Park YC (2008). Redox factor-1 mediates NF-kappaB nuclear translocation for LPS-induced iNOS expression in murine macrophage cell line RAW 264.7. Immunology.

[28] Angel P, Karin M (1991). The role of Jun, Fos and the AP-1 complex in cell-proliferation and transformation. Biochim. Biophys. Acta.

[29] Tseng CP, Ely BD, Pong RC, Wang Z, Zhou J, Hsieh JT (1999). The role of DOC-2/DAB2 protein phosphorylation in the inhibition of AP-1 activity. An underlying mechanism of its tumor-suppressive function in prostate cancer. J. Biol. Chem.

[30] Ozaki T, Habara K, Matsui K, Kaibori M, Kwon AH, Ito S, Nishizawa M, Okumura T (2010). Dexamethasone inhibits the induction of iNOS gene expression through destabilization of its mRNA in proinflammatory cytokine-stimulated hepatocytes. Shock.

[31] Cook T, Wang Z, Alber S, Liu K, Watkins SC, Vodovotz Y, Billiar TR, Blumberg D (2004). Nitric oxide and ionizing radiation synergistically promote apoptosis and growth inhibition of cancer by activating p53. Cancer Res.

[32] Qiu LQ, Sinniah R, Hsu SI (2004). Coupled induction of iNOS and p53 upregulation in renal resident cells may be linked with apoptotic activity in the pathogenesis of progressive IgA nephropathy. J. Am. Soc. Nephrol.

[33] Ding WX, Shen HM, Ong CN (2000). Critical role of reactive oxygen species and mitochondrial permeability transition in microcystin-induced rapid apoptosis in rat hepatocytes. Hepatology.

[34] Wei Y, Weng D, Li F, Zou X, Young DO, Ji J, Shen P (2008). Involvement of JNK regulation in oxidative stress-mediated murine liver injury by microcystin-LR. Apoptosis.

[35] Leiers T, Bihlmayer A, Ammon HP, Wahl MA (2000). [Ca(2+)](i)- and insulin-stimulating effect of the non-membranepermeable phosphatase-inhibitor microcystin-LR in intact insulin-secreting cells (RINm5F). Br. J. Pharmacol.

[36] Cnop M, Welsh N, Jonas JC, Jorns A, Lenzen S, Eizirik DL (2005). Mechanisms of pancreatic beta-cell death in type 1 and type 2 diabetes: many differences, few similarities. Diabetes.

[37] Prentki M, Segall L, Roche E, Thumelin S, Brun T, McGarry JD, Corkey BE, Assimacopoulos-Jeannet F (1998). Gluco-lipotoxicity and gene expression in the pancreatic beta cell. J Annu Diabetol Hotel Dieu.

[38] McDaniel ML, Corbett JA, Kwon G, Hill JR (1997). A role for nitric oxide and other inflammatory mediators in cytokine-induced pancreatic beta-cell dysfunction and destruction. Adv. Exp. Med. Biol.

[39] Shimabukuro M, Zhou YT, Levi M, Unger RH (1998). Fatty acid-induced beta cell apoptosis: A link between obesity and diabetes. Proc. Natl. Acad. Sci. USA.

[40] Maedler K, Sergeev P, Ris F, Oberholzer J, Joller-Jemelka HI, Spinas GA, Kaiser N, Halban PA, Donath MY (2002). Glucose-induced beta cell production of IL-1beta contributes to glucotoxicity in human pancreatic islets. J. Clin. Invest.

[41] Fischer A, Hoeger SJ, Stemmer K, Feurstein DJ, Knobeloch D, Nussler A, Dietrich DR (2010). The role of organic anion transporting polypeptides (OATPs/SLCOs) in the toxicity of different microcystin congeners *in vitro*: A comparison of primary human hepatocytes and OATP-transfected HEK293 cells. Toxicol. Appl. Pharmacol.

[42] Monks NR, Liu S, Xu Y, Yu H, Bendelow AS, Moscow JA (2007). Potent cytotoxicity of the phosphatase inhibitor microcystin LR and microcystin analogues in OATP1B1- and OATP1B3-expressing HeLa cells. Mol. Cancer Ther.

[43] Giacomini KM, Huang SM, Tweedie DJ, Benet LZ, Brouwer KL, Chu X, Dahlin A, Evers R, Fischer V, Hillgren KM (2010). Membrane transporters in drug development. Nat. Rev. Drug Discov.

[44] Chen W, Song L, Ou D, Gan N (2005). Chronic toxicity and responses of several important enzymes in Daphnia magna on exposure to sublethal microcystin-LR. Environ. Toxicol.

